# 

**Published:** 1904-07-23

**Authors:** 


### NOTICE TO CORRESPONDENTS.

All MSS., Letters, Books for Review, and other matters intended for the Editor should be addressed The Editor, "The Hospital," The Hospital Buildings, 28 & 29 Southampton Street, Strand, W.C.

The Editor cannot undertake to return rejected MS., even when accompanied by stamped directed envelope.

**Notice to Advertisers and Subscribers.**

All Advertisements, Orders for Copies of The Hospital, and Business Communications should be addressed to **THE MANAGER (Not to the Editor),** The Hospital, 28 & 29 Southampton Street, Strand, London, W.C.

The Cost of Subscription, post free, is as follows:—Per week, 3½d.; per quarter, 3s. 3d.; per half-year, 6s. 6d.; per annum, 13s. Foreign and Colonial:—Per annum, 19s. 6d., payable, in all cases, in advance.

**NOTICE.—Vol. XXXV. of "The Hospital" (October 1903 to March 1904), in handsome cloth binding, is now on sale at the office of this Journal, price 10s. 6d. Cases for binding the Numbers contained in this Volume 2s. Index to Vol. XXXV., 3½d., post free.**

**The Monthly part for July is now ready. Price 1s., by post, 1s. 3d.**

Sir John Brickwood has raised £1,000 towards new buildings at the Royal Portsmouth Hospital, and the chairman, Sir John Baker, has offered £500 for the same purpose. At the meeting, when the subject was discussed, the scheme to replace the old wards met with the usual opposition which is always forthcoming whenever radical improvements are suggested. Fortunately, however, in this instance the idea of patching up the old wards was negatived in favour of complete reconstruction.

The Board of Education have issued their regulations in regard to the education of defective and epileptic children. Special grants per unit will be made to certified schools holding classes for the purpose and to special schools. In the case of epileptic children the schools must provide board, lodging, and medical treatment.

### Medical Appointments.

**Charles Blair**, M.D.Durh., F.R.C.S.Eng., Surgeon to the Western Ophthalmic Hospital, London. **E. N. de Vere Dawson**, L.R.C.P. and S.Edin., L.F.P.S.Glasg., Additional Surgeon of Kimberley, Cape Colony, resident at Warrenton. **J. L. Dickie**, M.B., C.M.Aberd., Medical Officer and Public Vaccinator to the Fetteresso Parish Council. **Wynne Griffith**, L.R.C.P. and S.Edin., Medical Officer for the Pwllheli District of the Pwllheli Union. **S. J. J. Kirby**, M.D.Brux., L.R.C.P.Edin., M.R.C.S.Eng., Medical Officer to the Oulton Broad Urban District Council. **C. A. Kitching**, M.R.C.S., L.R.C.P.Lond., District Surgeon at Mossel Bay, Cape Colony. **F. C. Shaw**, M.B., M.Ch.Syd., Government Medical Officer and Vaccinator at Wyalong, N.S.W. **William Sutherland**, M.B., Ch.B., Resident Surgeon, Tuapeka Hospital, Lawrence, N.Z. **C. S. Sutton**, M.B., Ch.B.Melb., Additional Anæsthetist, Women's Hospital, Melbourne. **C. Gerrard Taylor**, M.D.Cantab., D.P.H., Medical Officer of Health of the Urban District of Finchley. **Arthur Francis Voelcker**, M.D., F.R.C.P.Lond., Examiner in Medicine, Society of Apothecaries. **F. E. A. Webb**, M.R.C.S., L.R.C.P.Lond., District Medical Officer of the Parish of Cambridge.

**"The Hospital" Institutional Library.**

**"Hospitals and Asylums of the World." 4 Vols., with a Portfolio of Plans, complete £12 12s.**

**"Burdett's Hospitals and Charities, 1904." 5s. net.**

**"Burdett's Official Nursing Directory." 8s. net.**

**"Cottage Hospitals, General, Fever, and Convalescent." 10s. 6d.**

**"Uniform System of Accounts for Hospitals and Public Institutions." New Edition. 4s. net.**

**"Hospital Expenditure: The Commissariat." 2s. 6d.**

**"A Legal Handbook for the Use of Hospital Authorities." 1s. 6d.**

**"The Cottage Hospital Case Book or Register of Patients." 10s. and 12s. 6d.**

**All these are published by the Scientific Press, Ltd., and may be obtained through any bookseller, or direct from the publishers, 28 and 29 Southampton Street, Strand, London, W.C.**

### TWO HELPS IN PRIVATE NURSING.

*JUST OUT.*

THE CHEAPEST CHARTS ISSUED.

### THE POCKET-BOOK OF TEMPERATURE CHARTS.

Containing 17 Twelve-Hour and 8 Four-Hour Charts, bound up in book form, and perforated so that each Chart may be removed after use.

Price **6d.** net; post free, **7d.**

THE BEST

### CASE-BOOK FOR PRIVATE NURSING.

Arranged for Day and Night Nurse's Report, with rulings in accordance with what has been found in practice to be the most suitable and approved System for Private Nurses, &c. Size of Book, $7\frac{1}{2}$ in. by $9\frac{1}{2}$ in., containing 48 pp., at the price of an Ordinary Exercise Book.

**3d.** net; **$4\frac{1}{2}$d.** post free.

### USEFUL NURSING HANDBOOKS.

### 1/- SERIES.

**FEVERS AND INFECTIOUS DISEASES:**
Their Nursing and Practical Management.
By William Harding, M.D.Ed.

**HOME NURSING OF SICK CHILDREN.**
By J. D. E. Mortimer, M.B., F.R.C.S.

**THE CARE OF CONSUMPTIVES.**
By W. H. Daw, M.R.C.S., L.R.C.P.Lond.

**NOTES ON PHARMACY AND DISPENSING FOR NURSES.**
New and Revised Edition.
By C. J. S. Thompson.

**THE MIDWIVES' POCKET BOOK.**
By Honnor Morten.

**THE EXAMINATION OF THE URINE.**

By J. K. Watson, M.D. **1s. 1d.** post free.

### 6d. SERIES.

**ON PREPARATION FOR OPERATION IN PRIVATE HOUSES.**
By Stanmore Bishop, F.R.C.S.

**MODERN NURSING IN PRIVATE PRACTICE.**
By Sir William Bennett, K.C.V.O., F.R.C.S.
**7d.** post free.

**THE SCHOTT TREATMENT FOR CHRONIC HEART DISEASES.**
By Richard Greene, F.R.C.P.Ed.

**ON SOME ASPECTS OF MEMORY.**

By John Aikman, M.D.

**SANATORIUM TREATMENT OF CONSUMPTION.**
By T. N. Kelynack, M.D., M.R.C.P. **7d.** post free.

**"SIMPLEX" DISTRICT NURSES' CASE-BOOK.**
Containing space for 50 Cases, Ruled and Printed.
Bound in Cloth Boards. **7d.** post free.

**Complete Catalogue of Nursing Manuals post free on application.**

**THE SCIENTIFIC PRESS, LTD.,** 28 & 29 Southampton Street, Strand, LONDON, W.C.

